# Characterization of the major surface glycoconjugates of *Trypanosoma theileri*

**DOI:** 10.1016/j.molbiopara.2023.111591

**Published:** 2023-08-29

**Authors:** Rupa Nagar, Isobel Hambleton, Michele Tinti, Mark Carrington, Michael A.J. Ferguson

**Affiliations:** a Wellcome Centre for Anti-Infectives Research, The School of Life Sciences, University of Dundee, Dundee DD1 5EH, United Kingdom; b Department of Biochemistry, University of Cambridge, Tennis Court Road, Cambridge CB2 1QW, United Kingdom

**Keywords:** *Trypanosoma theileri*, Glycoconjugate, Glycoinositolphospholipids, Glycosylphosphatidylinositol, Mucin, Mass-spectrometry

## Abstract

*Trypanosoma theileri* maintains a long-term extracellular infection with a low parasitaemia in bovids. The surface of this parasite is predicted to be decorated with several surface molecules including membrane surface proteases (MSPs), trans-sialidases and *T. theileri* putative surface proteins (TTPSPs). However, there are no experimental data to verify this hypothesis. Here, we have purified and partially characterized the surface glycoconjugates of *T. theileri* using biochemical and mass spectrometry-based approaches. The glycoconjugates fall into two classes: glycoproteins and glycolipids. Proteomic analysis of the glycoprotein fraction demonstrated the presence of MSPs and abundant mucin-like TTPSPs, with most predicted to be GPI-anchored. Mass spectrometric characterization of the glycolipid fraction showed that these are mannose- and galactose-containing glycoinositolphospholipids (GIPLs) that are larger and more diverse than those of its phylogenetic relative *T. cru*z*i*, containing up to 10 hexose residues and carrying either alkylacyl-phosphatidylinositol or inositol-phospho-ceramide (IPC) lipid components.

## Abbreviations

BSF: blood stream form
GIPLs: glycoinositolphospholipids
GPI: glycosylphosphatidylinositol
GC-MS: gas chromatography-mass spectrometry
ES-MS: electrospray mass spectrometry
Gal: Galactose
Man: Mannose
Glc: Glucose
GlcN: glucosamine
PI: phosphatidylinositol
Cer: ceramide
AAG: alkyl-acylgly-cerol
2-AEP: 2-aminoethylphosphonate
EtNP: ethanolamine phosphate
βGalf: β-galactofuranose
TMS: trimethylsilyl
TFA: trifluoroacetic acid

## 1 Introduction

The class Kinetoplastidea are unicellular protozoans that are evolutionarily divergent. The order Trypanosomatida within this class contains many parasitic species that colonise a variety of hosts. Some trypanosomatid species are pathogenic to humans and domestic animals, such as *Trypanosoma brucei* that causes human and animal African trypanosomiases, *T. cruzi* that causes American trypanosomiasis, or Chagas’ disease, and *Leishmania* spp. that cause visceral, cutaneous and mucocutaneous leishmaniases. These pathogenic trypanosomes have been studied extensively and represent some of the best characterized unicellular eukaryotic organisms [1,2].

Less well characterized are trypanosomatids of the subgenus Megatrypanum such as *T. theileri* which infects bovids, and has been found across Asia, Europe, Africa and America [3]. *T. theileri* is transmitted by contaminated faeces of tabanid flies and gains entry into the bovid host (Bovidae: cattle, buffalo, yak and some antelopes) through skin breaks or via contamination of the oral mucosa [4]. This organism is an extracellular parasite that maintains a low parasitaemia (~100 organisms/ml) inside the host and generally does not cause disease, unless the host’s immune system is compromised [5].

Protozoan parasites generally change their cell surface molecular architectures during their life cycles such that their cell surfaces match their needs for survival and infectivity in different biological environments. For the trypanosomatids characterized to date, the abundant surface molecules are mostly species- and life cycle stage-specific glycosylphosphatidylinositol (GPI) anchored glycoproteins, free GPI molecules (also known as glycoinositolphospholipids (GIPLs)) or GPI-anchored oligosaccharides, like *Crithidia fasciculata* lipoarabinogalactan (LAG) or GPI-anchored phospho-oligosaccharides, i. e., the *Leishmania* lipophosphoglycans (LPGs). These molecules have been variously implicated in protection, immune evasion, host- and vector-cell interactions. The structures and functions of trypanosomatid surface glycoconjugates are reviewed in [6–9].

Currently, there are no experimental data on the surface molecules of *T. theileri*. However, genomic and transcriptomic analyses suggest that its major surface molecules include membrane surface proteases (MSPs) (also found in *T. brucei* spp., *T. cruzi* and *Leishmania* spp.), transsialidases (also found in *T. cruzi* and *T. brucei* spp.), and four distinct families of *T. theileri* putative surface proteins (TTPSPs). The latter are predicted to encode mostly *O*-glycosylated GPI-anchored surface glycoproteins that are distinct from (at the amino acid sequence-level) but similar to (at the feature level) the mucin-like surface glycoproteins of *T. cruzi*. Importantly, the *T. theileri* genome does not appear to contain any variant surface glycoprotein (VSG) genes that are typical of *T. brucei* [1,10]. Overall, these genomic and transcriptomic features suggest that the surface glycoproteins of *T. theileri* may be most similar to those of *T. cruzi* [1]. What cannot be easily determined from these data, however, is what other non-proteinaceous surface molecules may be present. For example, in *T. cruzi* epimastigotes, free GPI molecules (most often referred to as glycoinositolphospholipids or GIPLs) are the most abundant surface molecules (~10 million copies per cell) alongside the GPI-mucins (~2 million copies per cell) [11].

Here, we have started to characterize the surface molecules of *T. theileri* at the biochemical level. We have applied the same differential solvent extraction and hydrophobic interaction chromatography protocols, developed by Almeida and colleagues [12,13] to those used to isolate the GPI-mucin and GIPL fractions of *T. cruzi*. As a positive control, we processed epimastigotes of *T. cruzi* Silvio X10/7 strain alongside tissue culture forms of *T. theileri* Edinburgh strain and so also report on the surface molecules of the Silvio X10/7 strain of *T. cruzi* for the first time.

## 2 Materials and methods

### 2.1 Cell culture and harvesting

Epimastigotes of *Trypanosoma cruzi* Silvio X10/7 A strain subclone A1 [14] were grown at 28 °C in RTH medium supplemented with 10 % heat-inactivated foetal calf serum (HI-FCS), filtered through 0.22 μm membrane [15,16]. Cultures were diluted 5–10 fold every 3–4 days. Cells (2 × 10^9^) were harvested at 2700 x g for 10 min at 4 °C and washed twice with ice cold trypanosome dilution buffer (TDB; 20 mM Na_2_HPO_4_, 2 mM NaH_2_PO_4_, 80 mM NaCl, 5 mM KCl, 1 mM MgSO_4_, 20 mM glucose, pH 7.7). The cell pellet was resuspended in 250 μl of MilliQ water with 1X protease inhibitor cocktail (Roche EDTA-free) and lysed with 3 cycles of freeze-thaw on dry ice in 70 % ethanol. The frozen cell lysate was freeze dried overnight. The dried pellet was stored in −20 °C until further use.

The *T. theileri* cells used in these experiments were a cloned population from *T. theileri* Edinburgh and were cultured in vitro [5]. Cells (5 × 10^8^) were harvested and washed twice with ice cold TDB, snap-frozen on dry ice and stored at −80 °C as a wet-sample until further use. At the same time, another 5 × 10^8^ cells were processed as described above, snap-frozen on dry ice and freeze dried overnight to produce a dry-sample. Both the dry and wet samples of *T. theileri* were used for glycoconjugates extraction.

### 2.2 Extraction and purification of glycoconjugates (mucins and GIPLs)

*T. cruzi* and *T. theileri* glycoconjugates were extracted from wet and/or dry cell samples using organic solvent extraction as previously described [13]. Briefly, the cells were resuspended in 250 μl of chloroform / methanol / water (1:2:0.8, by volume) and placed in sonicating water bath for 30 min. After sonication, the samples were centrifuged and the insoluble material was re-extracted with a further 250 μl of chloroform / methanol / water (1:2:0.8, by volume). The final insoluble pellet was used as a source of delipidated cellular material. The chloroform/methanol/water soluble fractions were pooled and evaporated under nitrogen. The residue was partitioned between 200 μl butan-1-ol and 100 μl water. The upper butan-1-ol phase saturated with water, containing the lipid fraction (F1) was collected, and the lower aqueous phase (F2 fraction) was washed twice with butan-1-ol saturated with water. The delipidated cellular materials were extracted three times by sonication with 250 μl 9 % butan-1-ol in water and the soluble material was pooled (F3 fraction). The carbohydrate-containing contents of the F1, F2 and F3 fractions were analysed by running 20 % of each fraction on an SDS-PAGE gel and staining with periodate-Schiff stain as described earlier [17]. The extracted glycoconjugates were further purified by hydrophobic interaction chromatography using the octyl-Sepharose CL-4B (Sigma) packed into a 12 cm × 1 cm i.d column. The F2 and F3 fractions were pooled, evaporated under nitrogen, and resuspended in 0.5 ml of 0.1 M ammonium acetate in 5 % propan-1-ol (v/v) (buffer A) and fractionated on an octyl-Sepharose column previously equilibrated in buffer A. The column was washed with 1 column volume of buffer A and eluted with a linear gradient from over 100 ml at a flow rate of 12 ml/h, starting with buffer A and ending with 70 % (v/v) propan-1-ol in water. The purity of each fraction was checked on a SDS-PAGE using the periodate-silver staining as described earlier [18].

### 2.3 Protease digestion of the glycoconjugate-rich F3 fraction

20 % of the *T. cruzi* and *T. theileri* (dry) F3 fractions were digested with 1 mg/ml Pronase in 20 mM ammonium bicarbonate, 5 mM calcium chloride, at 37 °C overnight. The digestion was checked on SDS-PAGE using periodate-Schiff staining.

### 2.4 Proteomic analysis of glycoconjugate-rich F3 fraction

To perform in solution trypsin digestion, 10 % of the F3 fractions of *T. cruzi* and *T. theileri* (dry) were dried and resuspended in 15 μl of 30 mM ammonium bicarbonate containing 5 M urea and 40 mM dithiothreitol, the mixture was incubated for 40 min at room temperature. Then, 5 μl of 100 mM iodoacetamide was added to S-alkylate the samples and incubated for 30 min in the dark. The samples were then diluted with 30 μl of 30 mM ammonium bicarbonate and digested twice with trypsin (0.012 μg each time) at 30 °C first overnight, then for another 6 h. After digestion, the samples were dried using vacuum centrifugation at 30 °C and digested peptides were resuspended in 50 μl, 1 % formic acid. The digested samples were analysed by liquid chromatography-tandem mass spectrometry (LC-MS^2^) on an LTQ Orbitrap Velos-Pro (Thermo Scientific) mass spectrometer coupled with a Dionex Ultimate 3000 RS (Thermo Scientific). Samples of 10 μl of each digested sample was loaded at 5 μl/min onto a trap column (PepMap nanoViper C18 column, 100 μm × 2 cm, 5 μm, 100 Å, Thermo Scientific) preequilibrated in buffer A (2 % acetonitrile and 0.1 % formic acid in Milli-Q water (v/v)) for 17 min. The trap column was washed with buffer A for 6 min at 5 μl/min and then the trap column was switched inline with a Thermo Scientific, resolving C18 column (PepMap RSLC C18 column, 75 μm × 50 cm, 2 μm, 100 Å) kept at a constant temperature of 50 °C. The peptides were eluted from the column at a constant flow rate of 300 nl/min with a linear gradient from 5 % to 35 % buffer B (80 % acetonitrile and 0.08 % formic acid in Milli-Q water (v/v) within 124 min, and from 35 % to 98 % buffer B in 2 min. The column was then washed for 20 min at 98 % buffer B and re-equilibrated in 2 % buffer B for 17 min. LTQ-Orbitrap Velos Pro was operated in data dependent positive ionization mode. The source voltage was set to 2.50 KV and the capillary temperature was 250 °C. A scan cycle comprised a high-resolution MS^1^ scan (*m/z* range from 335 to 1800) in the Orbitrap Velos-Pro followed by 15 sequential data-dependant MS^2^ scans using collision induced dissociation (CID) with threshold value set at 5000, the minimum injection time 200 ms, default charge state 2, isolation width 2 (*m/z*), normalised collision energy 35, activation Q 0.25, activation time 10 ms. The resolution of the Orbitrap Velos was set to 60,000 after accumulation of 1,000,000 ions. Precursor ion charge state screening was enabled, with all unassigned charge states and singly charged species rejected. The lock mass option was enabled for survey scans to improve mass accuracy.

### 2.5 Proteomic data analysis and bioinformatics

The LC-MS^2^ proteomic data for the F3 fraction of *T. theileri* was searched with MaxQuant Version 1.5.8.3 against the version 39 of the *T. theileri* Edinburgh isolate genome downloaded from TriTrypDB [19]. The parameters used were Trypsin/P digestion and Oxidation (M) and Acetyl (Protein N-term) as variable modifications. Other default parameters were left untouched. The proteinGroups.txt output file of MaxQuant was parsed to filter out: Only identified by site (n = 16), Reverse (n = 29) Potential contaminant (n = 26) protein groups and protein groups with less than 2 unique peptides identified (n = 475). The protein identification numbers of the leading proteins of the MaxQuant protein groups were used for further analysis. The protein identification numbers for the families TTPSP1 (n = 556), TTPSP2 = (n = 304), TTPSP3 (n = 145) and TTPSP4 (n = 61) were retrieved from column B of supplementary Table 2 in Kelly et al., 2017 [1] using the family accession numbers OG0000004, OG0000013, OG000003 and OG0000108. Protein identification numbers were converted to those of version 39 of the *T. theileri* Edinburgh isolate genome using TriTrypDB. Protein identification numbers of MSP (also known as GP63) protein families were retrieved from TriTrypDB using the GP63 keyword for version 39 of the *T. theileri* Edinburgh isolate genome (n = 200). Protein GPI anchor attachment sites were predicted with big-PI [20] and PredGPI [21]. PredGPI predictions were retained only with a false discovery rate less than 0.01. Proteins were considered to have a GPI anchor if one or both predictors provided a positive hit. SignalP-5.0 was used to predict the proteins with a signal peptide [22]. The processed molecular weight of the proteins without a signal peptide and GPI anchor signal peptide (using the predicted omega site of predGPI) was computed with biopython [23]. Serine plus Threonine and Proline frequencies of the *T. Theileri* proteome, mucins and F3 fraction were computed with a custom script.

### 2.6 Carbohydrate composition and myo-inositol analysis of glycoconjugate fractions by GC-MS

Aliquots of 5 % of each octyl-Sepharose fraction from fraction 19–28 were mixed with an internal standard of 0.5 nmol of *scyllo*-inositol and 0.5 nmol of D_6_-*myo*-inositol (1,2,3,4,5,6-d6 *myo*-Inositol- from QMX, Labs, Thaxted, UK); of this mixture 90 % was used to perform GC-MS monosaccharide composition analysis and remaining 10 % was used for *myo*-inositol analysis, as described in [24]. GC–MS was performed on Agilent Technologies, 7890B Gas Chromatography system with 5977 A MSD equipped with Agilent J&W HP-5 ms GC Column (30 m x 0.25 mm, 0.25 μm) with He carrier gas at 0.5 ml/min. For monosaccharide composition analysis, the dried samples were subjected to methanolysis at 85 °C for 4 h, followed by re-N-acetylation and TMS (trimethylsilyl) derivatization prior to GC-MS. The temperature program started at 95 °C (for 1 min) then to 140 °C at 30 °C/min and then to 265 °C at 5 °C/min and held at 265 °C for 5 min. Electron impact mass spectra (EI-MS) were collected by linear scanning over *m/z* 50 – 650. Each sugar was quantified by integrating the total ion-current chromatograms and using empirically determined the molar relative response factors (MRRFs) from co-analysed standard mixtures. For *myo*-inositol analysis, the samples were subjected to strong acid treatment using 6 N HCl at 110 °C for 24 h, followed by drying and TMS derivatization. Selected ion monitoring (SIM) GC-MS analysis, as described in [13,24] was used. The total *myo*-inositol content in each fraction was quantified by comparing the areas of the *m/z* 318 (sample) and *m/z* 321 (internal standard) ions.

### 2.7 ES-MS analysis of T. cruzi epimastigote and T. theileri GIPL fractions

Aliquots (10 %) of the octyl-Sepharose purified *T. cruzi* GIPL fraction (fraction 32) and *T. theileri* GIPL containing fractions were pooled, evaporated and dissolved in 50 μl of 50 % propan-1-ol and, using the static infusion nanoflow probe tips (M956232AD1-S, Waters), infused into the source of a Thermo LTQ Orbitrap XL mass spectrometer (for *T. cruzi* GIPLs) and Thermo LTQ Orbitrap Velos Pro mass spectrometer (for *T. theileri* GIPLs). The data for *T. cruzi* GIPL MS and MS^2^ were collected in positive ion mode whereas the data for *T. theileri* GIPL MS and MS^2^ were collected in both positive and negative ion mode. The precursors with expected GIPL species with [M+2 H]^2+^ *m/z* values were subjected to tandem MS^2^ using collision induced dissociation (CID). The capillary voltage was 1.2 KV and CID 30 V was used for MS^2^ of the precursor ions.

## 3 Results and discussion

### 3.1 Extraction and purification of T. theileri glycoconjugates

Throughout these studies we have used *T. cruzi* epimastigotes as a convenient control for glycoconjugate extraction, purification and characterization. Ideally, we would have used *T. cruzi* bloodstream form trypomastigotes as a control, but these are hard to obtain in the quantities needed.

**Fig. 1:**
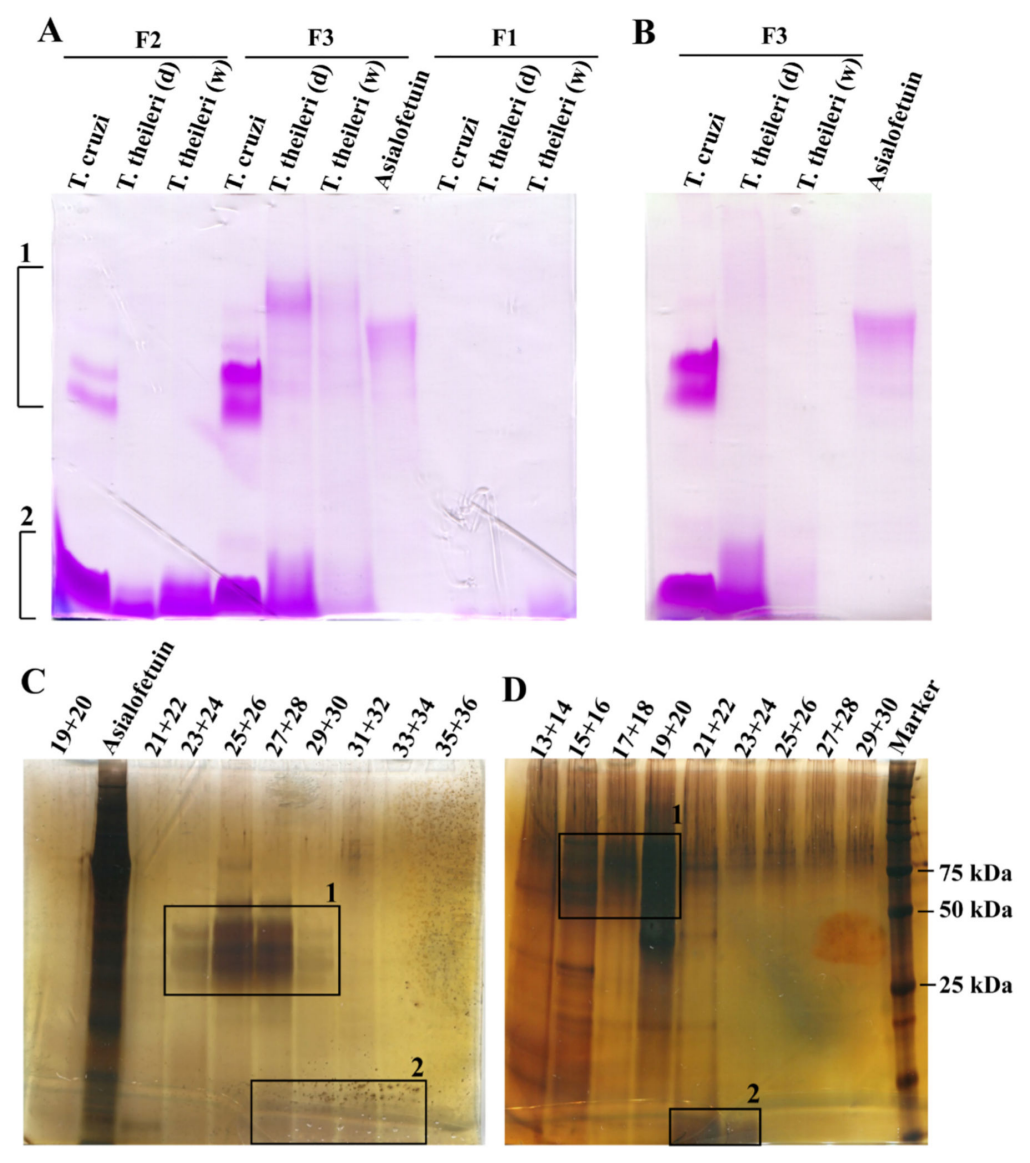
SDS-PAGE analysis of extracted and purified glycoconjugates of *T. theileri* and *T. cruzi*. **(A)** SDS-PAGE and periodate-Schiff staining of F1, F2 and F3 fractions (see Scheme 1) from dry *T. cruzi* and wet (w) and dry (d) *T. theileri* cell samples, as indicated. **(B)** SDS-PAGE and periodate-Schiff staining of F3 fractions after Pronase digestion at 37 °C for 12 h. A sample of 10 μg of asialofetuin was also loaded as a positive control for staining. **(C and D)** show SDS-PAGE and periodate-silver staining of octyl-Sepharose fractions of the combined F2 and F3 fractions from *T. cruzi* and *T. theileri*, respectively. The fraction numbers analysed are as indicated. The last lane in (**D**) shows molecular weight markers. Boxes indicate GPI-mucins/glycoproteins (1) and GIPLs (2). SDS-PAGE and periodate-silver staining of all *T. cruzi* and *T. theileri* octyl-Sepharose fractions eluted are shown in Fig. S1A and Fig. S1B, respectively. Asialofetuin (10 μg) was run on each gel as a positive control for staining.

**Scheme 1:**
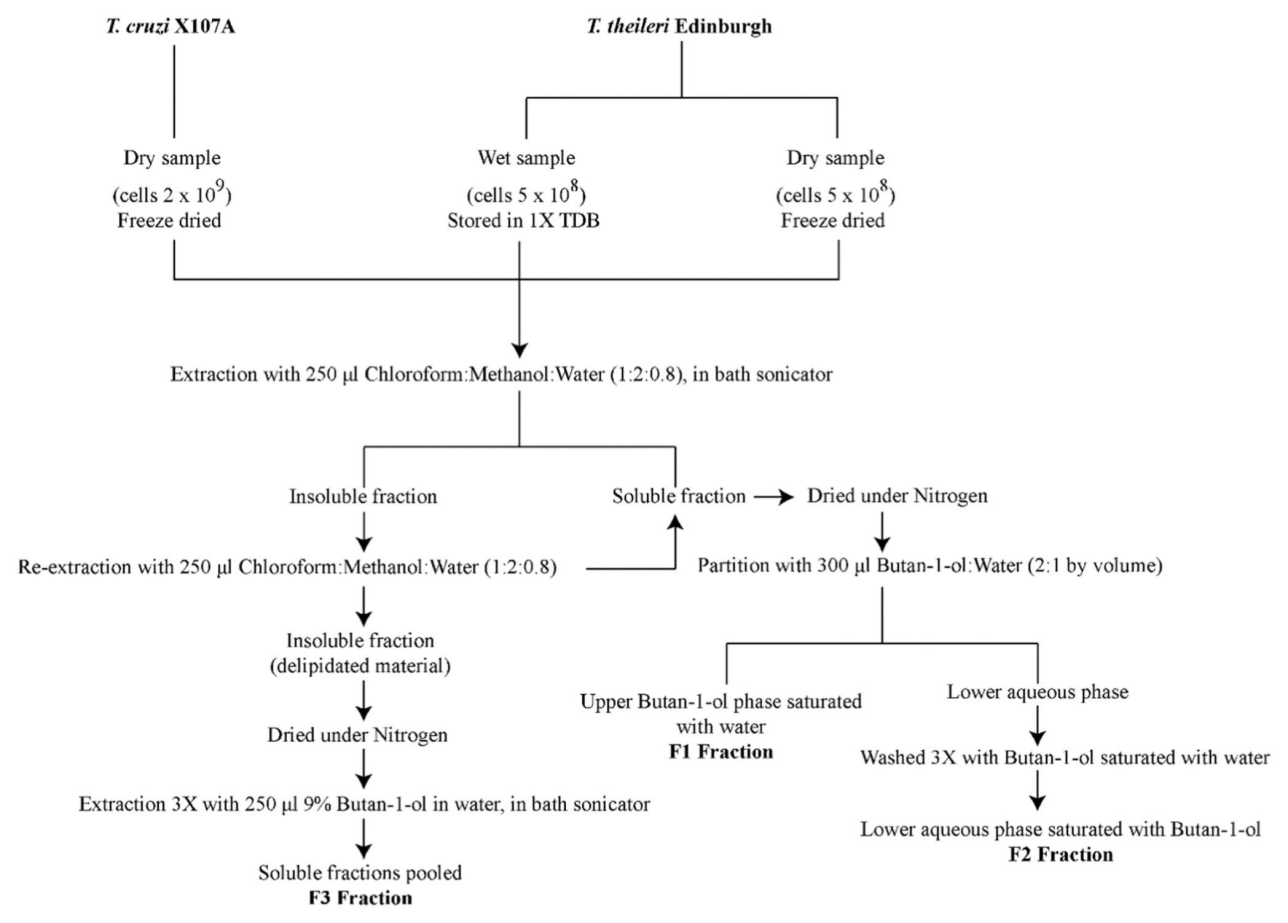
Differential solvent extraction procedure to enrich GIPLs and GPI-mucins glycoconjugates. The schematic flowchart elaborates the solvent extraction process [12,13] used to extract glycoconjugate fractions from *T. cruzi* and *T. theileri* cells..

*T. theileri* BSF and *T. cruzi* epimastigotes were grown in culture and their respective glycoconjugates were extracted using a solvent extraction method previously optimised for the extraction of *T. cruzi* GPI-anchored mucins and GIPLs (Scheme 1) [13]. As expected, most of the GIPLs were recovered in the F2 fraction while the F3 fraction contained both glycoproteins (GPI-mucins in the case of *T. cruzi*) and GIPLs, as observed by SDS-PAGE and periodate-Schiff staining for carbohydrate (Fig. 1A). The extraction was performed on freeze-dried cells for *T. cruzi* and on both freeze-dried and wet cells for *T. theileri*. The latter gave a better yield of GIPLs in F2 but a poorer yield of glycoproteins in the F3 fraction.

The *T. theileri* glycoproteins have higher apparent molecular weights than the *T. cruzi* epimastigote mucins. Digestion of the *T. cruzi* and *T. theileri* (dry) F3 fractions with Pronase showed that the *T. theileri* glycoproteins bands are sensitive to Pronase, confirming their proteinaceous nature. The relative resistance of the *T. cruzi* GPI-mucins to Pronase is consistent with their known high-density of O-glycosylation [25] (Fig. 1B).

The F2 and F3 fractions from the *T. cruzi* and *T. theileri* (wet and dry) extractions were combined and fractionated using a 5–70 % propan-1-ol gradient on an octyl-Sepharose column and the eluted glycoconjugates were identified by SDS-PAGE and periodate-silver staining (Fig. 1C and 1D, respectively). *T. theileri* glycoproteins and GIPLs (glycoproteins in fractions 15–20 and GIPLs in fraction 21–24, Fig. 1D and Fig. S1B) were recovered at a lower percentage of propan-1-ol than the corresponding *T. cruzi* mucins and GIPLs (GPI-mucins in fractions 23–30 and GIPLs in fraction 27–34, Fig. 1C and Fig. S1A), suggesting that the *T. theileri* glycoconjugates are more polar than those of *T. cruzi* and/or that their lipidic components are less hydrophobic. With respect to the *T. theileri* glycoproteins, additional polarity could be due to the size and polarity of the protein component and/or the extent of glycosylation. The extraction and chromatographic characteristics of the *T. theileri* glycoproteins are otherwise reminiscent of *T. cruzi* GPI-mucins, particularly of the larger *T. cruzi* GPI-mucins from bloodstream trypomastigote [26].

To further characterize the *T. theileri* glycoprotein and GIPL fractions, the former was analysed by proteomics and the latter by GC-MS and ES-MS.

### 3.2 Proteomic analysis of T. theileri glycoprotein fraction

**Fig. 2:**
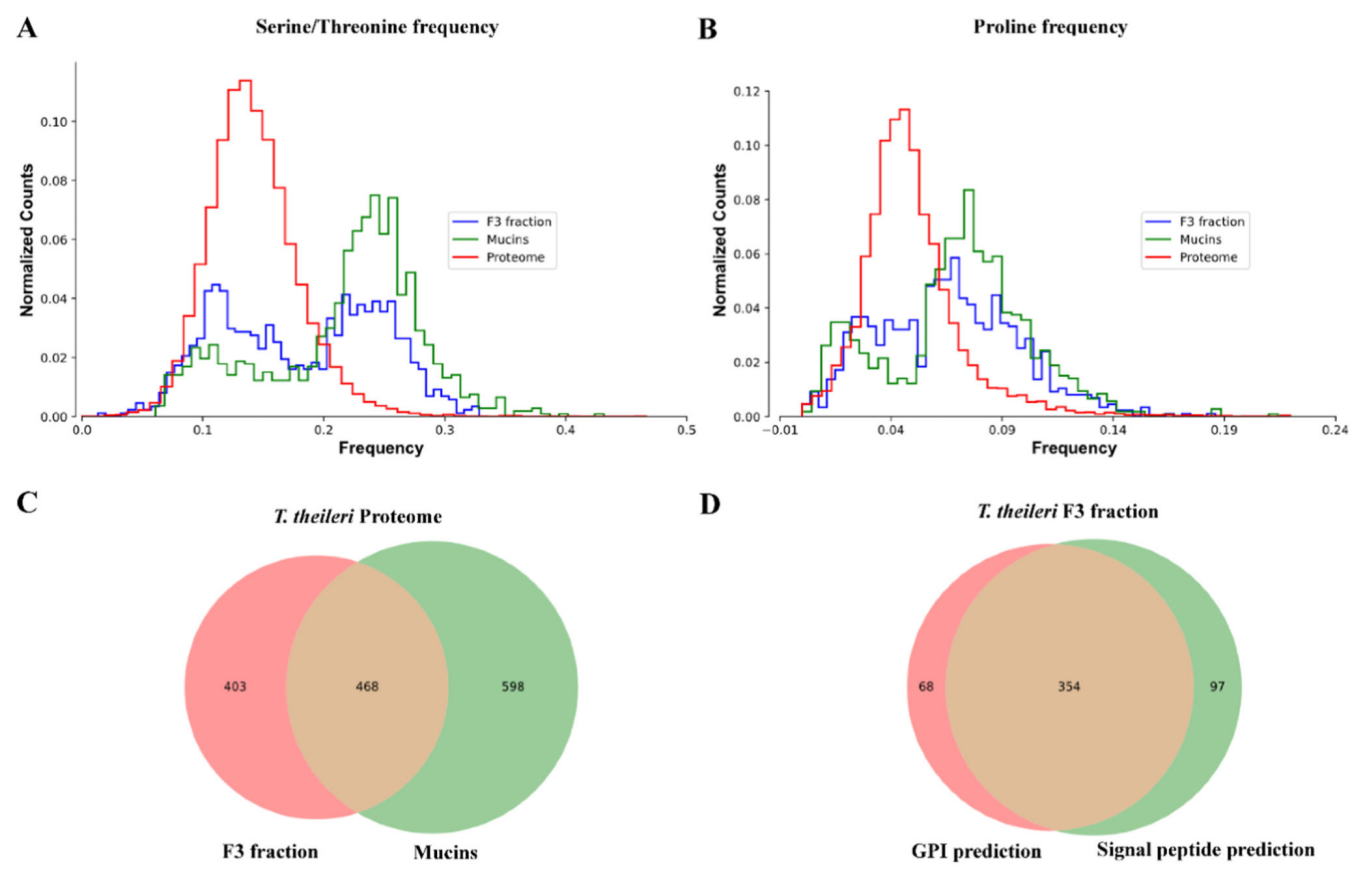
Comparisons of *T. theileri* total, TTPSPs and the F3 fraction proteomes. **(A)** Normalized counts (y-axis) of the Serine/Threonine frequencies (x-axis) for the proteins identified by LC-MS in the *T. theileri* F3 fraction (blue), for the TTPSPs (mucin) annotated proteins (green) and for the whole *T. theileri* proteome (red). The counts are normalized so that the sum of the bars heights is one. **(B)** Normalized counts (y-axis) of the Proline frequencies (x-axis) for the proteins identified by LC-MS in the *T. theileri* F3 fraction (blue), for the TTPSPs (mucin) annotated proteins (green) and for the whole *T. theileri* proteome (red). **(C)** Venn diagram showing the overlap of proteins identified in the *T. theileri* F3 fraction by mass spectrometry (n = 871) and the proteins annotate as TTPSPs (mucins) in the *T. theileri* proteome (n = 1066). **(D)** Venn diagram showing the overlap of proteins identified in the *T. theileri* F3 fraction with predicted GPI anchor signal sequences (GPI prediction) (n = 422) and predicted N-terminal signal peptides (signal peptide prediction) (n = 451).

The F3 fraction of *T. theileri* (dry) was subjected to reduction, alkylation and trypsin digestion and the resulting peptides were analysed by LC-MS^2^, 871 proteins were identified by searching against the protein sequence database of *T. theileri* Edinburgh available at Tri-TrypDB [19]. Of the 871 proteins identified, 468 belonged to one of the four TTPSPs orthogroups, and 4 were identified as MSPs (Table 1 and Fig. 2C) [1].

The predicted TTPSP amino acid sequences have highly conserved N-terminal signal sequences and C-terminal GPI-addition sequences and are predicted to be Ser, Thr and Pro-rich (typical of *O*-glycosylated regions) near the C-terminus of the mature polypeptide. We compared the Ser, Thr and Pro frequencies of the entire theoretical *T. theileri* Edinburgh strain proteome, the theoretical TTPSP1–4 sub-proteome and of the experimentally determined F3 fraction proteome (Fig. 2). This showed that Ser/Thr and Pro frequencies of the F3 fraction are more similar to those of the TTPSPs sub-proteome to those of the total proteome (Fig. 2A and B). Moreover, we found that 347 out of the 468 identified TTPSP proteins are predicted to have both an N-terminal signal peptide and a GPI anchor signal peptide (Fig. 2D and Table 1).

Overall, these data provide the first experimental evidence that a large number of predicted TTPSP genes are simultaneously expressed in a *T. theileri* in vitro culture and that the majority of these gene products are likely to be transported into the ER by virtue of their predicted cleavable N-terminal signal peptides, and attached to GPI membrane anchor precursors in the ER by virtue of their predicted GPI anchor signal peptides.

### 3.3 Inositol and monosaccharide analysis of the T. theileri GIPL fraction

Aliquots of fractions 16–25 obtained from the octyl-Sepharose hydrophobic interaction chromatography column (Fig. 1D) were subjected to *myo*-inositol analysis and to monosaccharide analysis (Fig. S2) by GC-MS. The components *myo*-inositol, mannose and galactose were detected above background in fractions 21–25 with an overall molar ratio of 1: 4: 9, a composition consistent with the presence of GIPLs.

### 3.4 ES-MS analysis of T. cruzi and T. theileri GIPLs

**Fig. 3:**
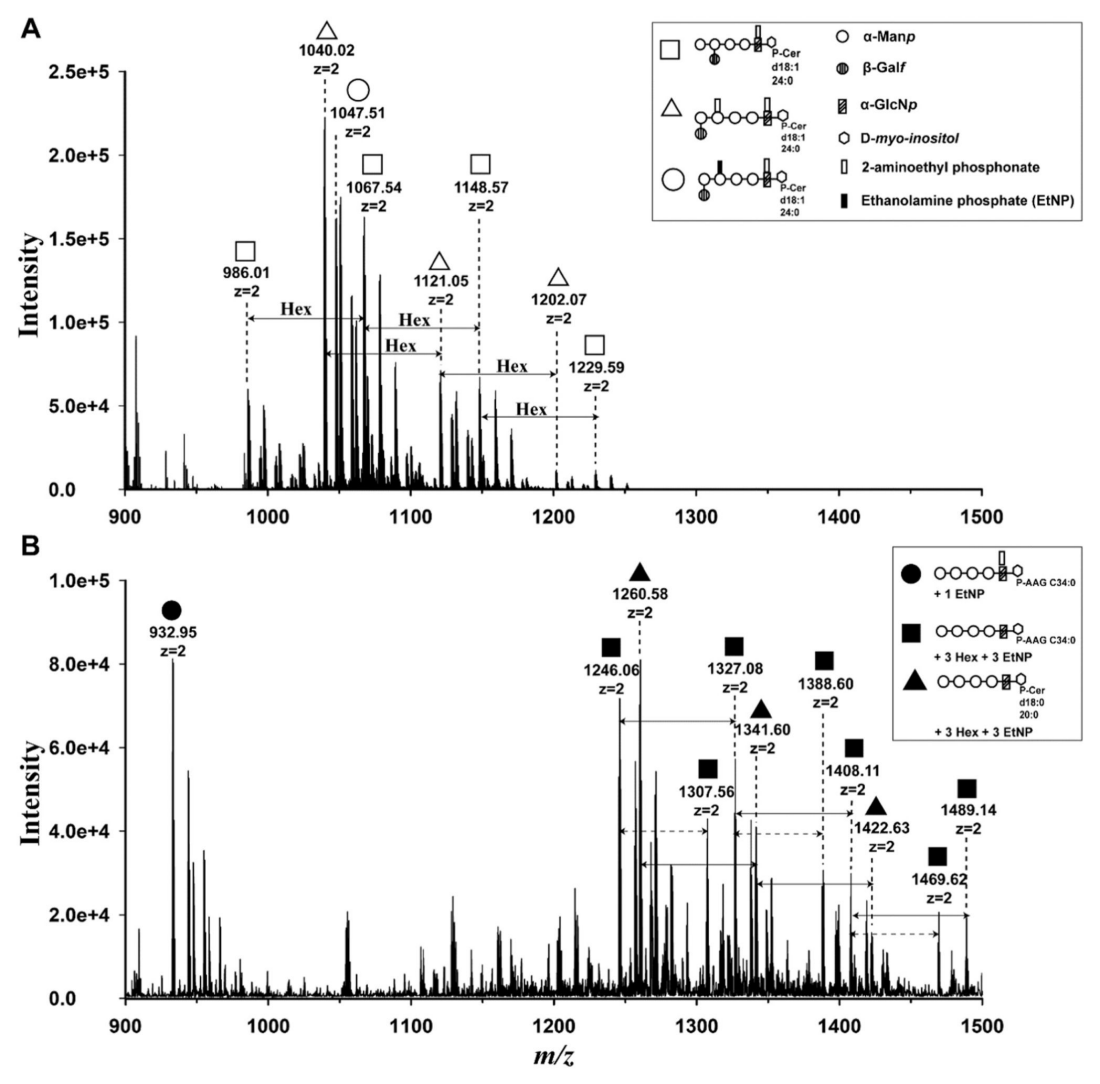
ES-MS analysis of *T. cruzi* X10/7 and *T. theileri* Edinburgh strain GIPLs. **(A)** Positive ion ES-MS spectrum of *T. cruzi* GIPLs. The putative molecular species (inset) are assigned according to their accurate *m/z* values in comparison with published data [22,23]. Three molecular series differing by hexose units are annotated by open boxes, triangles, and circles (refer to Table 2). **(B)** Positive ion ES-MS spectrum of *T. theileri* GIPLs. The GIPL species at *m/z* 932.95 annotated as a solid circle and series starting as *m/z* 1246.06 annotated with solid boxes, are alkyl-acylglycerol (AAG) based lipids, whereas the series starting at *m/z* 1260.58, annotated with solid triangles, are ceramide (Cer) based lipids. Both major series contain at least 7 hexoses and 3 EtNP molecules (refer to Table 2). In **(B)** solid arrows represent the hexose addition, whereas the dashed arrows represent the EtNP addition. **Note:** The MS spectra shown here are annotated with the highest abundance isotopic *m/z* values, whereas true monoisotopic *m/z* values (observed from close inspection of the spectra) are shown in (Table 2).

One of the GIPL containing fractions of *T. cruzi* Silvio X10/7 epimastigotes (Fig. 1C, fraction 32) was concentrated and analysed by ES-MS and ES-MS^2^ in positive ion mode as a positive control. The individual GIPL molecular species were observed principally as [M+2H]^2+^ ions, although [M+2Na]^2+^ and/or [M+H+Na]^2+^ ions were also present. Based on previous reports on *T. cruzi* GIPLs [27–31], we were able to assign putative molecular species to the observed ions (Table 2, Fig. 3A). The MS^2^ data of the two main molecular species at *m/z* 1040.02 (monoisotopic value 1039.51, Table 2) and *m/z* 1067.54 (monoisotopic value 1067.04, Table 2) are shown in Fig. S3A and Fig. S3B, respectively. These spectra are consistent with both species containing inositol-phosphoceramide (IPC) components containing sphingosine (C18:1) long-chain base with C24:0 fatty acid. Our data are broadly consistent with a more detailed study on the GIPLs of Silvio X10/1 epimastigotes [27], except in that study sphinganine (C18:0) was the principle long chain base and additional hexoses (beyond a single Gal*f* residue) were not described. Indeed, *T. cruzi* epimastigote GIPLs have only been previously described with a maximum of two hexose (Gal*f*) residues in addition to their Man_4_-GlcN cores [31], whereas the X10/7 GIPLs reported here appear to contain up to 4 additional hexoses. Finally, the ES-MS data (Fig. 3A) suggest that the X10/7 sample contains species with either one or two 2-aminoethylphosphonate (2-AEP) substituents, as well as species with one 2-AEP and one ethanolamine phosphate (EtNP) as described in [27,31]. The subtle differences in the molecular species observed here for Silvio strain X10/7 compared with previously published work in X10/1 are most likely due to differences in strains and culture conditions.

The GIPL fraction of BSF *T. theileri* Edinburgh strain (Fig. 1D, fractions 21–24) were pooled, concentrated and analysed by ES-MS and ES-MS^2^ as described above. The ES-MS data were acquired in both positive (Fig. 3B) and negative ion (Fig. S4) mode. The individual GIPL molecular species were observed principally as [M+2H]^2+^ ions, with some [M+2Na]^2+^ adducts, in positive ion mode and as [M-2H]^2-^ ions in negative ion mode. The *T. theileri* GIPLs ES-MS spectrum contained two major [M+2H]^2+^ ion molecular series, starting at *m/z* 1246.06 (monoisotopic value 1245.56, Table 2) and *m/z* 1260.58 (monoisotopic value 1260.07, Table 2), further modified by up to 3 additional hexose residues and up to 1 additional EtNP residue (Fig. 3B, Table 2). Additionally, another small molecular species at *m/z* 932.95 (Table 2) was also observed. A 14.5 a.m.u (29 Da) difference between the adjacent members of the two major ion series (*m/z* 1246.06, annotated with solid squares and *m/z* 1260.58 annotated as triangles in Fig. 3B) suggested the presence of similar headgroups with different lipid species. This was confirmed by subjecting these major molecular ions to MS^2^ fragmentation using CID. Based on the MS^2^ fragmentation (Fig. S5) we were able to assign the putative molecular species to observed *m/z* ions (Table 2). The *m/z* 932.95 and *m/z* 1246.06 ions, upon CID fragmentation, released an ion at *m/z* 565.55 corresponding to an alkyl-acylglycerol (AAG) lipid with a total C34:0 (O-C16:0/18:0) hydrocarbon content (Fig. S5A **and** Fig. S5B). The *m/z* 1260.58 ion, upon CID fragmentation, released an ion at *m/z* 594.58 corresponding to an inositol-phosphoceramide (IPC) lipid components with C38:0;O3 composition (most likely with a C18:0 sphinganine long-chain base and an hydroxy-C20:0 acyl chain and/or a C18:0 phytosphingosine long-chain base and a C20:0 acyl chain) (Fig. S5C).

Based on the accurate mass data in the ES-MS and MS^2^ fragmentation spectra (Fig. S5), we suggest that the *m/z* 1246.06 ion and its relatives (marked with solid squares in Fig. 3B, Table 2) are alkyl-acylglycerol (AAG) lipid with a total C34:0 hydrocarbon content (O-C16:0/18:0) based GIPLs species and contain 7–10 hexose residues and 3–4 EtNP substituents. Whereas the species at *m/z* 1260.58 and its relatives (marked with solid triangles in Fig. 3B, Table 2) are inositol-phosphoceramide (IPC) lipid based GIPLs species that contain 7–9 hexose residues and 3 EtNP substituents. The species at *m/z* 932.95 which is a AAG based lipid contains 4 hexose residues and one EtNP and one 2-AEP substituent. Interestingly, the GIPLs species observed for *T. theileri* are significantly larger than known *T. cruzi* GIPLs in terms of the total number of hexose residues (up to 10 in total for *T. theileri* as opposed to up to 8 for *T. cruzi*) and EtNP residues (up to 4 in total for *T. theileri* as opposed to 1 EtNP and 1 or 2 2-AEP for *T. cruzi*).

### 3.5 Conclusions

Taken together, these data support the conclusion that the majority of surface glycoconjugates of *T. theileri* are GPI-anchored mucin-like glycoproteins (encoded by all four classes of TTPSP genes [1]), most similar to the GPI-mucins of *T. cruzi* bloodstream form trypomastigotes [26], interspersed with abundant and relatively large GIPL glycophospholipids, and some GPI-anchored MSPs. The ES-MS data shows that *T. theileri* GIPLs are larger and more structurally diverse than those of its phylogenetically close relative *T. cruzi*. Although the structural characterization done in this study is not complete, the partial characterizations provide a good general framework for considering that *T. theileri* has a cell surface molecular architecture more similar to that of *T. cruzi* than to other well-characterized kinetoplastids, like *T. brucei* and the *Leishmania*. A potential limitation of this study is that the methods of extraction and fractionation used may have prevented the identification of less polar surface glycoproteins.

## Supplementary Material

Supplementary Material

## Figures and Tables

**Table 1:** Characteristics of the proteins identified in the F3 fraction. Showing the number of proteins identified in the F3 fraction of *T. theileri* by protein class and the number of those proteins with a predicted N-terminal signal peptide (SigP) and GPI anchor signal peptide (GPI) and the number with both types of signal peptide (Both). The percentage of proteins with both types of signal peptide is also recorded (Both, %). The median amino acid sequence lengths and median protein molecular weights (MW) of the processed proteins without the predicted N-terminal and GPI anchor signal peptides (but without glycosylation) are also shown.

Protein Class	No. of proteins identified in F3	SigP	GPI	Both	Both, %	Median sequence length (aa)	Median MW of proteins (kDa)
MSP	4	4	3	3	75	606	67.98
TTPSP1	272	241	234	207	76.1	269	27.19
TTPSP2	120	88	110	81	67.5	261	26.92
TTPSP3	43	38	41	37	86	348	39.03
TTPSP4	33	32	22	22	66.7	224	24.33
OTHER	399	48	12	4	1	194	21.02

**Table 2:** Compositions of *T. cruzi* Silvio X10/7 strain and *T. theileri* Edinburgh strain GIPLs deduced from ES-MS and ES-MS^2^ analyses.

Species	Major [M+2H1^2+^ monoisotopic ions observed	theoretical *m/z* values	molecular formula	MW of native molecule	Proposed GIPL species assignment	
					Substituted glycans	Lipid component
***T. cruzi* Silvio 10/7**	^□^986.0106	986.0088	C86H161O42N3P2	1970.0030	[2-AEP]-[Hex_5_GlcNIno*P*]^a^	Cer-C18:1 sphingosine- C24:0 fatty acid
	^▵^1039.5174	1039.5156	C88H167O44N4P3	2077.0166	[2-AEP]_2_-[Hex_5_GlcNIno*P*]^b^	Cer-C18:1 sphingosine- C24:0 fatty acid*
	^○^1047.5144	1047.5130	C88H167O45N4P3	2093.0155	[EtNP][2-AEP]- [Hex_5_GlcNIno*P*]^c^	Cer-C18:1 sphingosine- C24:0 fatty acid
	^□^1067.0364	1067.0352	C92H171O47N3P2	2132.0558	[2-AEP]-[Hex_6_GlcNIno*P*]^a+^	Cer-C18:1 sphingosine- C24:0 fatty acid*
	^▵^1120.5440	1120.5420	C94H177O49N4P3	2239.0694	[2-AEP]2- [Hex_6_GlcNIno*P*]^b+^	Cer-C18:1 sphingosine- C24:0 fatty acid
	^○^1128.5418	1128.5394	C94H177O50N4P3	2255.0643	[EtNP][2-AEP]- [Hex_6_GlcNIno*P*]^c+^	Cer-C18:1 sphingosine- C24:0 fatty acid
	^□^1148.0633	1148.0616	C98H181O52N3P2	2294.1086	[2-AEP]- [Hex_7_GlcNIno*P*]^a++^	Cer-C18:1 sphingosine- C24:0 fatty acid
	^▵^1201.5699	1201.5684	C100H187O54N4P3	2401.1223	[2-AEP]2- [Hex_7_GlcNIno*P*]^b++^	Cer-C18:1 sphingosine- C24:0 fatty acid
	^□^1229.0903	1229.0880	C104H191O57N3P2	2456.1615	[2-AEP]- [Hex_8_GlcNIno*P*]^a+++^	Cer-C18:1 sphingosine- C24:0 fatty acid
***T. theileri*** **Edinburgh**	^●^932.95224	932.94733	C77H148O41N3P3	1863.88011	[EtNP][2-AEP]- [Hex_4_GlcNIno*P*]^1^	AAG-C34:0 *
	^◼^1245.56476	1245.52828	C97H184O60N4P4	2489.04202	[EtNP]_3_- [Hex_7_GlcNIno*P*]^2^	AAG-C34:0 *
	^▴^1260.07820	1260.04156	C98H187O60N5P4	2518.06857	[EtNP]_3_- [Hex_7_GlcNIno*P*]^3^	Cer C38:0;O3 *
	^◼^1307.06792	1307.03255	C99H190O63N5P5	2612.05055	[EtNP]_4_- [Hex_7_GlcNIno*P*]^4^	AAG-C34:0
	^◼^1326.59049	1326.55470	C103H194O65N4P4	2651.09484	[EtNP]_3_- [HexgGlcNIno*P*]^2+^	AAG-C34:0
	^▴^1341.10579	1341.06797	C104H197O65N5P4	2680.12139	[EtNP]_3_- [Hex_8_GlcNIno*P*]^3+^	Cer C38:0;O3
	^◼^1388.09601	1388.05896	C105H200O68N5P5	2774.10337	[EtNP]_4_- [Hex_8_GlcNIno*P*]^4+^	AAG-C34:0
	^◼^1407.61875	1407.58111	C109H204O70N4P4	2813.14767	[EtNP]_3_- [Hex_9_GlcNIno*P*]^2++^	AAG-C34:0
	^▴^1422.13115	1422.09438	C110H207O70N5P4	2842.17422	[EtNP]_3_- [Hex_9_GlcNIno*P*]^3++^	Cer C38:0;O3
	^◼^1469.12214	1469.08537	C111H210O73N5P5	2936.15620	EtNP]_4_- [Hex_9_GlcNIno*P*]^4++^	AAG-C34:0
	^◼^1488.64594	1488.60752	C115H214O75N4P4	2975.20049	[EtNP]_3_- [Hex_10_GlcNIno*P*]^2+++^	AAG-C34:0

The table shows the *m/z* values of the major [M+2H]^2+^ ions observed by ES-MS analysis (observed) alongside theoretical values for the atomic compositions, predicted monoisotopic MWs and molecular species compositions for the substituted glycans and alkyl-acylglycerol (AAG) or ceramide (Cer) lipid components of the GIPLs. The square, triangle and circle symbols are to correlate the species in Fig. 3.

* Indicates that the lipid identity was confirmed by MS^2^.

a,b,c Series in *T. cruzi*

1,2,3 *T. theileri* GIPLs refer to GIPL series that have a common lipid component but differ in number of 2-AEP/EtNP substituents attached to the glycan. The number of additional hexose units within a series are indicated by the number of + symbols.

## Data Availability

The mass spectrometry proteomics data have been deposited to the ProteomeXchange Consortium via the PRIDE [32] partner repository with the dataset identifier PXD029837.
