# Supplementary material for: Characterization of the major surface glycoconjugates of Trypanosoma theileri
Source: Mol Biochem Parasitol. Author manuscript; Available in PMC 2025 Sep 23. (PMC7618162; doi:10.1016/j.molbiopara.2023.111591)
Supplement: Supplementary Material [file EMS208333-supplement-Supplementary_Material.docx]

Characterization of the major surface glycoconjugates of

*Trypanosoma theileri*

Rupa Nagar^a^, Isobel Hambleton^b^, Michele Tinti^a^, Mark Carrington^b*^, Michael A. J. Ferguson^a*^

^a^ *Wellcome Centre for Anti-Infectives Research, The School of Life Sciences, University of Dundee, Dundee DD1 5EH, United Kingdom*

*^b^ Department of Biochemistry, University of Cambridge, Tennis Court Road, Cambridge CB2 1QW, United Kingdom*

* Corresponding authors, Email: [mc115@cam.ac.uk](mailto:mc115@cam.ac.uk) [m.a.j.ferguson@dundee.ac.uk](mailto:m.a.j.ferguson@dundee.ac.uk),


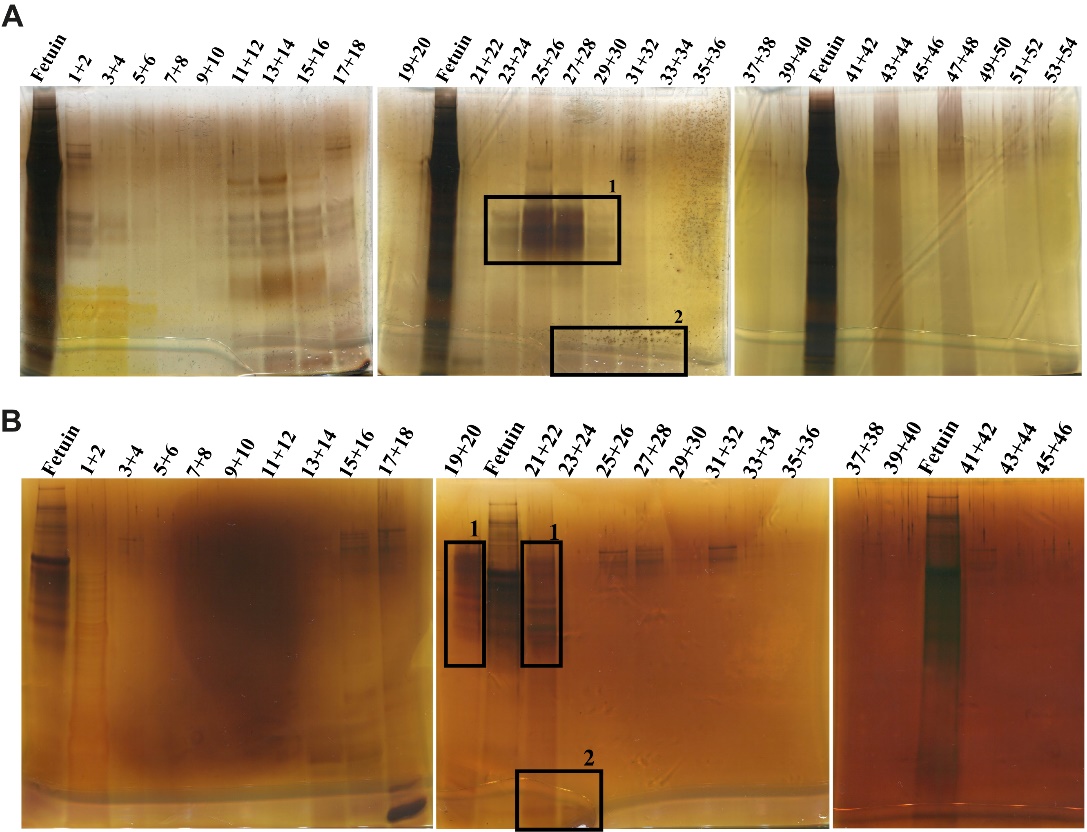


**Fig. S1: octyl-Sepharose (OS) fractionation of *T. cruzi* and *T. theileri* surface glycoconjugates:** Silver-periodate staining profile of *T. cruzi* glycoconjugates (**A**) and *T. theileri* (dry) (**B**) obtained after OS fractionation. The lanes are marked as the OS fraction number. Boxes indicate GPI-mucins/glycoproteins (1) and GIPLs (2).





**Fig. S2: Carbohydrate and *myo*-inositol analysis of *T. theileri* glycoconjugates by GC-MS:** The graph represents the total amount (in pmoles) of myo-inositol, mannose and galactose (y-axis) in each octyl-Sepharose purified fraction (x-axis).





**Fig. S3: ES-MS^2^ fragmentation of *T. cruzi* GIPLs species (corresponding to MS data shown in Fig. 3A).** ES-MS^2^ fragmentation of the major molecular species *m/z* 1040.02 (**A**) and *m/z* 1067.54 (**B**) observed in ES-MS^1^ analysis of *T. cruzi* GIPLs. MS^2^ was obtained using CID. Here, *m/z* 632.63 and *m/z* 614.62 represent the identified lipid species, Cer d18:1/24:0 whereas *m/z* 1446.39 in (**A**) and *m/z* 1339.38 in (**B**) represent the remaining glycan core without ceramide.





**Fig. S4: ES-MS of *T. theileri* GIPLs species in negative ion mode.** The lone GIPL species at *m/z* 930.93 annotated as a solid circle is a small alkyl-acylglycerol (AAG) based GIPL species (**Table 2**, corresponding to *m/z* 932.95). The series starting as *m/z* 1244.05 annotated with solid boxes, are alkyl-acylglycerol (AAG) based lipids (**Table 2**, corresponding to *m/z* 1245.56), whereas the series starting at *m/z* 1258.56, annotated with solid triangles, are ceramide (Cer) based lipids (**Table 2**, corresponding to *m/z* 1260.07). Both series contain at least 7 hexoses and 3 EtNP molecules (**Table 2**). The solid arrows represent the hexose addition, whereas the dashed arrows represent the EtNP addition.





**Fig. S5: ES-MS^2^** **fragmentation of *T. theileri* GIPLs species in positive ion.** The major molecular species observed in *T. theileri* GIPLs MS^1^ spectrum were subjected to MS^2^ to confirm the identity. (**A**) and (**B**) represent the MS^2^ CID fragmentation of the doubly charged parent ions observed at *m/z* 932.95 and *m/z* 1045.56, respectively. The daughter ion at *m/z* 565.55 in both (**A**) and (**B**) confirms the lipid component to be an alkyl-acylglycerol (AAG) having C34:0 composition. The solid arrows in (**B**) represent the Hex addition and dotted arrows represent the Hex subtraction from the parent ion. (**C**) represents the MS^2^ CID fragmentation of the parent ion observed at *m/z* 1260.07. The daughter ion at *m/z* 594.58 confirms the lipid component to be a ceramide Cer (C38:0;O3). The dotted arrows represent the Hex subtraction from parent ion. The identity of each lipid component has been confirmed by MS^2^ fragmentation in negative ion as well (data not shown) and is consistent with the pattern observed in positive ion.
